# Modelling the Economic Impact of Reducing Loneliness in Community Dwelling Older People in England

**DOI:** 10.3390/ijerph18041426

**Published:** 2021-02-03

**Authors:** David McDaid, A-La Park

**Affiliations:** Care Policy and Evaluation Centre, Department of Health Policy, London School of Economics and Political Science, London WC2A 2AE, UK; a.park@lse.ac.uk

**Keywords:** cost-effectiveness analysis, social activities, loneliness, older people

## Abstract

Loneliness has been associated with poor mental health and wellbeing. In England, a 2018 national strategy on loneliness was published, and public health guidelines recommend participation in social activities. In the absence of existing economic evidence, we modelled the potential cost effectiveness of a service that connects lonely older people to social activities against no-intervention. A 5-year Markov model was constructed from a health and social care perspective. Parameters were drawn from the literature, with the intervention structure based on an existing loneliness alleviation programme implemented in several settings across England. Univariate and probabilistic sensitivity analyses were undertaken. The total expected cost per participant in the intervention group is £7131 compared to £6783 in the usual care group with 0.45 loneliness free years (LFY) gained. The incremental cost per LFY gained is £768; in the probabilistic sensitivity analysis the intervention is cost saving in 3.5% of iterations. Potentially such interventions may be cost-effective but are unlikely to be cost-saving even allowing for sustained effects and cumulative adverse health and social care events averted. Empirical studies are needed to determine the cost-effectiveness of these interventions, ideally mapping changes in loneliness to the quality of life, in order to allow the key metric in health economic studies, cost per quality adjusted life year to be estimated.

## 1. Introduction

Loneliness can be thought of as a subjective, unpleasant, and distressing phenomenon resulting from a discrepancy between an individual’s desired and achieved levels of social relations [1]. Even though periods of loneliness for all of us are an inevitable part of life, there is increasing evidence suggesting that enduring loneliness potentially can have an adverse impact on mental and physical health across different age groups [2,3,4,5]. The risk of premature death may also be associated with more severe levels of loneliness in older people [6,7,8].

The policy interest in measures to reduce loneliness in England is high, with the creation of a ministerial post with a specific responsibility for loneliness, and publication of a national strategy to address the issue in 2018 [9]. The issue of loneliness, and potential impacts on mental health in particular, has also been highlighted by England’s Chief Medical Officer as one key adverse consequence of ongoing measures to enforce social distancing and minimise physical contacts during the current COVID-19 pandemic [10].

Loneliness has also been recognised as a risk factor for poor mental health and wellbeing by the National Institute for Health and Care Excellence (NICE), an independent authority that makes recommendations on appropriate public health interventions in England. Evidence on the effectiveness of approaches to tackle loneliness that include a strong element of socialisation and group activities is growing [11]. Current NICE guidelines to promote the mental health and wellbeing of older people include support for actions to both publicise, and if there is not enough existing provision, consider providing a range of group-based social activities that meet the needs and interests of people who have retired from work [12].

Potentially investment in programmes to address loneliness in older people through socialisation and group activities, if effective, may also be cost-effective from a public health perspective, promoting better physical and mental health and wellbeing across the population. However, there remains relatively little evidence on the cost-effectiveness and resource implications of interventions whose primary goal is to reduce loneliness [13].

In the absence of such empirical data from trials, health economic modelling studies are widely used to help determine the potential strength of the economic case for action [14]. Models essentially are mathematical frameworks that estimate the consequences of different policy and practice decisions. They can be used to bring together evidence on the effectiveness, resource use, and costs from multiple sources to help reduce the level of uncertainty for decision makers associated with an investment in any intervention. They can look at the likelihood of cost-effectiveness, that is whether the additional benefits of any action are worth the additional costs required to achieve them, under a wide range of conditions, varying many different assumptions on costs, uptake, effects, and outcomes.

One of the principal approaches is Markov modelling. It has the advantage of being able to model uncertain processes over multiple time periods known as cycles and reflect circumstances where individual health and outcomes can fluctuate. This means that Markov models can also be used for projecting longer-term costs and outcomes than may be practical in empirical studies, such as a very long-term follow up on the costs and benefits of a mental health treatment in older adults [15]. There are many other applications of these models in health economics. For instance, they have been used to look at the cost-effectiveness of different cancer treatments [16], where at different points in time individuals may be in different disease states, including remission, recurrence or death, as well as the economic case for preventive interventions such as screening programmes [17]. Markov modelling has also been used by public health agencies in England to help support local decision makers, for instance, to develop their mental health promotion and disorder prevention strategies, including work to prevent bullying in schools, suicide in adults, and poor mental health in older people [18].

Given the lack of information on the cost-effectiveness of loneliness alleviation programmes, this paper describes a decision-analytical Markov model that provides illustrative information on a service intended to alleviate loneliness in people aged 65 and older. The model synthesises short term costs and experience in tackling loneliness in England with information on the potential health and social care benefits that may be gained through a reduction in loneliness over a 5-year time period. The comparator in the model is assumed to be no intervention.

The service modelled is assumed to be similar to many local signposting services offered in a number of locations in England to help older people make new social connections in their community. As an illustrative example, we assume that our model intervention is similar to that offered by Reconnections, a personalised support and community response service to loneliness initially operated in Worcestershire in southern England since 2015 [19] and now also replicated in Surrey and London.

This service was created in 2015 by Social Finance, a not for profit organisation that strives to find better ways of tackling social problems in the UK. It is also the first service in the UK to be funded through a social impact bond to tackle loneliness. These bonds provide an investment to address social problems and look to fund preventative interventions. They link financial success to the delivery of measured social outcomes. If, and only if, the social outcome improves (in this case loneliness), the outcome payor repays the investors for their initial investment plus a return for the financial risks they took.

The service works with older people to understand their individual strengths and needs; rebuilding confidence and supporting them to connect with people, places, or activities in their community. For example, individuals may benefit from a focus on arts-related activity, sport and exercise, spirituality, connections with local communities or those with whom they share an identity, and/or on enhancing their online lives.

## 2. Materials and Methods

### 2.1. Model Structure

**Figure 1:**
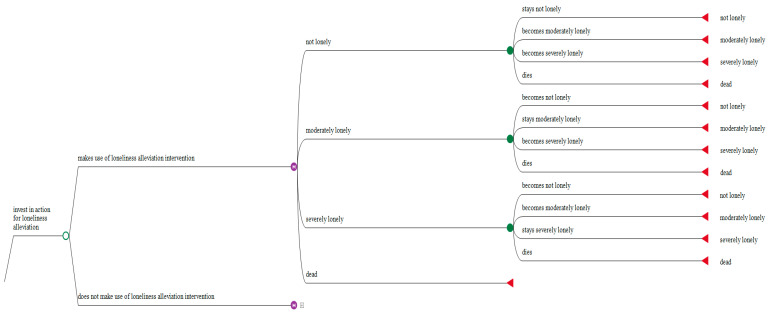
Excerpt of the Markov model.

A four-state Markov model has been constructed. Figure 1 illustrates the principal model pathways. The model runs over five cycles with each cycle lasting one year, comparing participation in the service against no intervention All individuals in the model initially are assumed to have moderate or severe levels of loneliness, reflecting the eligibility criteria in Reconnections, where only older people with initial scores between 7 to 9 (moderate loneliness) and 10 to 12 (severe loneliness) using the four-item version of the UCLA-Loneliness scale [20] were eligible for participation.

The model assumes, that in the first year of the model, the intervention group receives support for a period of between 6 and 9 months from a trained volunteer who helps identify opportunities to participate in a wide range of local community activities with the objective of reducing loneliness, as well as improving health and wellbeing. Beyond this time period, the aim is for service users to continue with the new social activities that they have been pursuing. After the first year, participants either continue to participate in their chosen activities (at their own cost) or may disengage from these activities. The model assumes that changes in loneliness achieved during the first year of support from Reconnections potentially can be sustained (to different extents) over a further 4 years if individuals continue to participate in community activities. To be conservative no further benefits are assumed to accrue to individuals who drop out of the programme.

The model then considers the potential impacts of participation in the service on time spent in one of four mutually exclusive different states: Not lonely, moderate lonely (lonely some of the time), severely lonely (lonely most of the time), and death for the intervention and no intervention groups. In each cycle, individuals can transition between each of these states, as well as into the finite death state.

Due to their social impact bond contractual obligations, Reconnections routinely assessed the loneliness scores of all their clients at a 1-year follow up. This meant that aggregate information on changes in loneliness outcomes for more than 1200 participants from this non-randomised case study were available to estimate transition probabilities, allowing us to make a preliminary estimate of the potential effectiveness of these programmes.

Looking at the outcomes, utility values used for the quality of life cannot currently be mapped to states of loneliness. Therefore, in this model, we simply assume that each cycle (year) spent in the state of not being lonely has a value of 1, while each cycle spent severely lonely has a value of 0. We assume that each cycle spent moderately lonely has a value of 0.5. The model then looks at the potential increased risks associated with different levels of loneliness in the intervention and no intervention groups for poor health and related need for additional health and social care service utilisation, compared with individuals who are not lonely in each cycle. Resource use and costs associated with differing risks of poor physical and mental health for different states of loneliness are also computed for each model cycle. We do not include additional costs related to death, assuming these to be the same for both intervention and non-intervention groups. Cumulative loneliness free time and total costs over the 5-year period are calculated for both groups. The incremental cost per additional hypothetical loneliness free year gained is then estimated.

Table 1 summarises parameters used in the model, assumptions on distributions used in the probabilistic sensitivity analysis (PSA), sources for data and transition probabilities. We have modelled potential additional health and social care costs for several conditions where a significant association between loneliness and a greater 1-year incidence of chronic illness has been reported. These include systematic reviews and meta-analyses that have reported an association between loneliness and major chronic health conditions, including stroke and coronary heart disease [5] and dementia [21]. A recent analysis of multiple waves of the English Longitudinal Survey on Ageing (ELSA) reports an association between loneliness at baseline and long-term higher levels of depression [4], while an analysis of the cross-sectional Adult Psychiatric Morbidity survey in England also suggests an association between both severe and moderate levels of loneliness and rates of intentional self-harm [22]. Loneliness has also been shown to be a risk factor for nursing home admission in logistic regression models making use of ELSA data [23]. In England, severe loneliness has been associated with a higher utilisation of primary care general practitioner (GP) services [24,25], as well as hospital accident and emergency services compared to the non-lonely [25]. A recent analysis of data from the Survey of Health, Ageing and Retirement in Europe (SHARE) also suggests a significant association between the greater use of GP services and being socially isolated [26].

In the model, with the exception of self-harm, we assume conservatively that any increased incidence in poor physical and mental health only applies to individuals who experience severe levels of loneliness. This may underestimate the benefits to the health and social care sectors of reducing moderate levels of loneliness, but insufficient data are available in the literature. We also assume that 44% of model participants initially meet the criteria for severe levels of loneliness based on the experience reported by Reconnections with their programme. For illustrative purposes in our baseline analysis, we assume that there would be a 19.5% reduction in individuals who received the intervention being either severely lonely or moderately lonely at a 1-year follow up based on the experience in Reconnections for individuals who had the most severe levels of loneliness at baseline. These figures must be treated cautiously given there is no counterfactual comparison data on individuals who declined to participate in Reconnections. Nonetheless, our model assumption may be conservative as almost 40% of individuals with moderate levels of loneliness at baseline in Reconnections had UCLA4 scores of 6 or less at a 1-year follow up and were no longer deemed lonely. Without intervention, we assume no change in the loneliness state. The annual sustained participation rate is assumed to be 89%, as seen with Reconnections over a 2-year period.

### 2.2. Analysis Perspective

Our primary analysis is conducted from a health and social care perspective. Health and social care service costs in England for physical and mental health conditions that may be associated with loneliness, including the greater use of primary and long-term residential care services, are shown in Table 1. The total budget for the service was divided by the number of participants that served to estimate the mean intervention cost per client [33]. Table 1 also includes the costs of additional informal care related to dementia and strokes. These impacts on informal care are included in our secondary analysis, along with the benefits from programme participants themselves becoming volunteers. We use a very conservative likelihood of participants becoming volunteers for 3 h per week derived from a community connection project in England [43]. All costs are reported in 2019 British pounds (£) with costs discounted at an annual rate of 3.5% and outcomes at 1.5%, a lower discount rate which can be used when assessing public health interventions [44].

It is important to always undertake sensitivity analysis as part of any economic modelling. This is due to the fact that there is usually uncertainty on the representativeness of many parameters in models, for instance, on the level of effectiveness of the intervention, or the costs associated with different health problems. A number of sensitivity analyses were conducted to assess the robustness of the analysis. This includes both the univariate sensitivity analysis of specific model parameters, where the model is run varying the values of key parameters one at a time, as well as probabilistic sensitivity analysis (PSA) using Monte Carlo simulation modelling In this Monte Carlo simulation, the uncertainty associated with the variables in Table 1 can be estimated as a probability distribution. This is done by randomly sampling a value for each of these parameters from within its probability distribution simultaneously and then calculating the incremental cost-effectiveness. We repeat this exercise 10,000 times. The range over which the parameters are varied will depend on the assumptions made on their potential distribution. Input variables were assigned beta, gamma, and log normal shaped distributions, as appropriate [45]. We can then visually show the results of this PSA on a cost-effectiveness plane, where all 10,000 combinations of cost and effect can be plotted (See Figure 4 in results section). All analyses are modelled using TreeAge Pro software (TreeAge Software, LLC, Williamstown, MA, USA).

## 3. Results

### 3.1. Summary Results

Summary results of the modelling analysis are presented in Table 2. In the base case scenario, the incremental cost per loneliness free year gained is £ 768. The total expected cost per participant in the intervention group over 5 years is £ 7131 compared to £ 6783 in the usual care group. Over this time period, 0.45 additional loneliness free years would be gained.

### 3.2. Univariate Sensitivity Analysis

**Figure 2:**
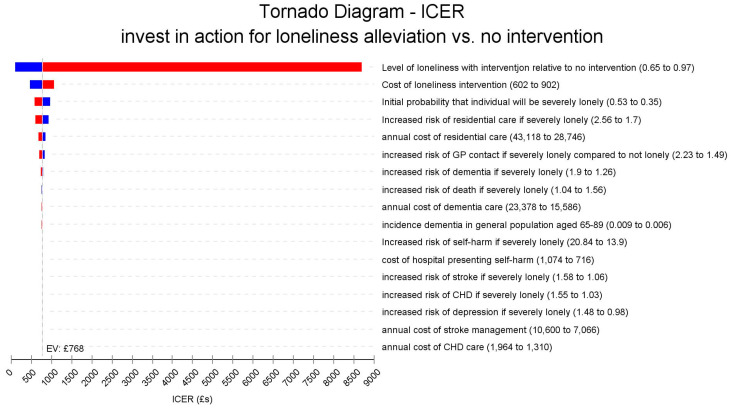
Tornado diagram of the univariate sensitivity analyses.

We examined how the results of our analysis change when we varied different individual variables in the model, one at a time, by up to 20% from their mean values in Table 1. The results are shown in Figure 2. This shows a “Tornado” diagram, so-called since it resembles a tornado with the most sensitive impacts of changing any variable shown at the top of the diagram and the least sensitive at the bottom. A vertical line shows the mean expected incremental cost effectiveness ratio (ICER) of £ 768 per loneliness free year gained in our base case scenario. The red bar segments indicate that the value of each parameter has increased, while the blue segments show that parameter values have fallen. Values to the right of the vertical base case scenario line indicate a less favourable cost-effectiveness with the cost per loneliness free year increasing compared to the base case scenario, while those to the left indicate an improvement in cost-effectiveness with the cost per loneliness free year gained reducing.

**Figure 3:**
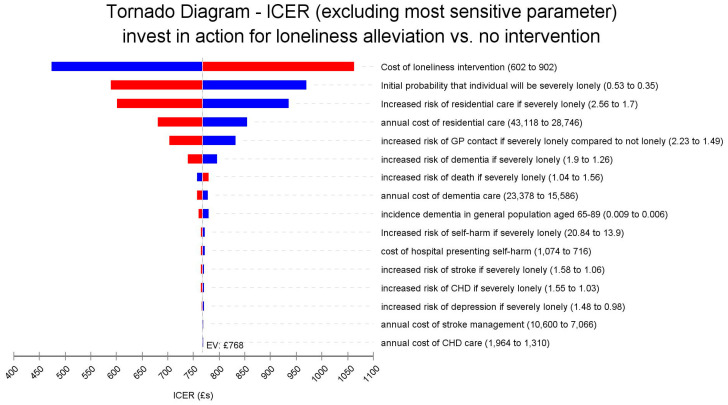
Tornado diagram of the univariate sensitivity analyses excluding the effectiveness parameter.

Figure 2 shows that the model is very sensitive to changes in the relative effectiveness of the intervention in reducing the likelihood of being severely lonely. If effectiveness is reduced by 10%, the ICER would become less favourable rising from £ 768 to £ 1856, while a 20% reduction in the effect would increase this to £ 8699. In contrast, a 20% improvement in effectiveness would lead to more favourable ICERs of £ 335 and just £ 96, respectively. The magnitude of impacts on ICER for all the other factors is much smaller. Figure 3 also shows the Tornado diagram, this time excluding the most sensitive effectiveness parameter. In this diagram, the cost of the intervention can be better seen to also have some impact. In our base case scenario the cost per participant is £ 752, if these costs could be reduced by 20% to £ 602 then the ICER becomes more favourable falling to £ 473, while a 20% increase in the intervention cost to £ 902 would mean a modestly less favourable ICER of £ 1062. As Figure 3 shows, changes in other factors including the relative risk of residential care if severely lonely, costs of residential care, and increased risk of GP contacts have very little impact on the ICER. In addition to the sensitivity analyses shown in Figure 2 and Figure 3, we also assessed the impact of setting the discount rate for outcomes at 3.5%, the same rate as for the costs. This would mean a marginally less favourable ICER of £ 805.

### 3.3. Probabilistic Sensitvity Analysis

**Figure 4:**
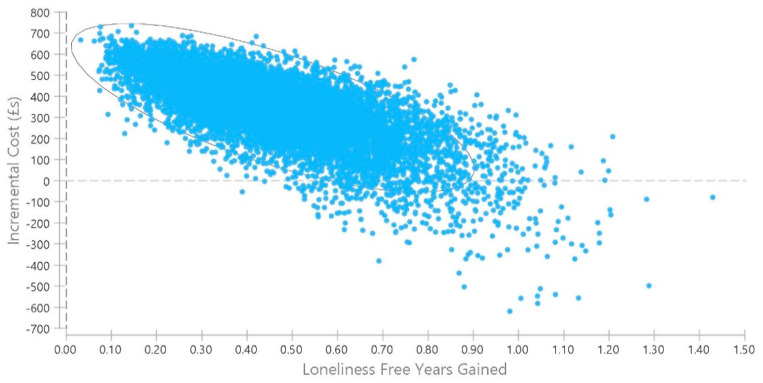
Probabilistic sensitivity analysis, loneliness alleviation versus no action.

A probabilistic sensitivity analysis was also performed, using Monte Carlo simulation with 10,000 replications sampled from the distributions presented in Table 1. The cost-effectiveness plane for incremental cost-effectiveness of the loneliness alleviation intervention compared to no action from a health and social care perspective is shown in Figure 4. The elliptical circle includes 95% of the simulations in the model. In just 3.5% of the simulated pairs of costs and outcomes is the intervention dominant over no action, that is with lower costs and better outcomes. In the remaining simulations it is more effective but more costly.

**Figure 5:**
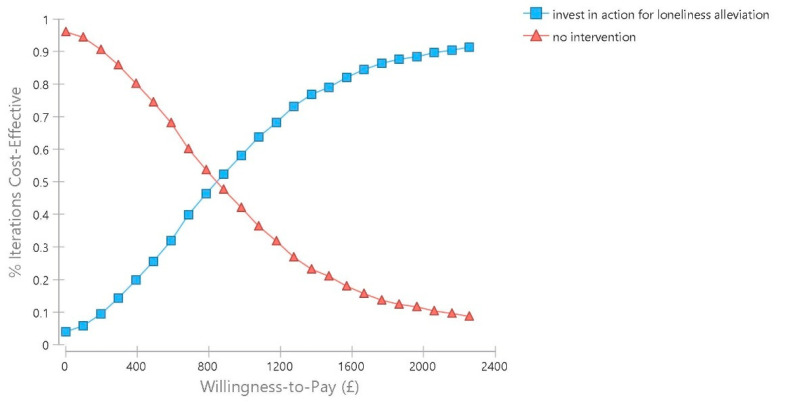
Cost-effectiveness acceptability curve.

We also constructed a cost-effectiveness acceptability curve. This indicates the probability that the intervention will be considered cost effective given different levels of willingness by policymakers to invest in measures to avoid loneliness up to a maximum of £ 2256, three times the value of the intervention cost. There is no accepted threshold for the additional time spent without being lonely, so this maximum willingness to pay value is for illustrative purposes. Ideally, future studies will map changes in levels of loneliness to levels of quality of life so that the commonly used metric in health economics of cost per quality adjusted life year gained can be calculated. As Figure 5 illustrates, for our health and social care perspective model, the intervention has a higher probability of being cost effective than no action if the willingness to pay reaches £ 836. At the willingness to pay levels of £ 1000, £ 1500, and £ 2000, respectively the probability of the intervention being considered cost effective is 59.8%, 79.7%, and 88.9%.

### 3.4. Alternative Scenario

Moreover, we modelled the intervention taking account of additional informal care costs associated with strokes [35] and dementia [30]. Furthermore, we included some very marginal economic benefits from participants of a loneliness alleviation programme themselves becoming volunteers. Table 3 shows the summary results under this scenario, with a lower ICER of £ 695. The total expected cost per participant in the intervention group over 5 years is £ 7878 compared to £ 7564 in the usual care group.

In probabilistic sensitivity analysis under this scenario, the intervention has a higher probability of being cost effective than no action if the willingness to pay level is at least £ 766. At the willingness to pay levels of £ 1000, £ 1500, and £ 2000, respectively the probability of the intervention being considered cost effective is 63.1%, 81.2%, and 90.4%. (See Supplementary for Figure S1 showing the additional cost-effectiveness plane and Figure S2 with the cost-effectiveness acceptability curve for this scenario).

## 4. Discussion

There is a considerable policy interest in addressing loneliness as a public health concern. In England, a cross-governmental national strategy was launched in 2018 to address the issue [9]. The NICE public health guidelines also recommend participation in group-social activities to tackle social isolation and loneliness as a way of promoting the mental wellbeing and independence of older people [12].

Reviews of evaluations of interventions that aim to tackle loneliness and social isolation continue to highlight the need to strengthen what is known about their effectiveness, [46,47]. Randomized controlled trial evidence on support to facilitate social connection and reduce loneliness remains very limited or set in very different country contexts [11,48,49]. Many of the approaches used to address loneliness, at least in a UK context, are provided and funded by non-governmental organisations, often on shoestring budgets, meaning that any evaluations undertaken tend to be minimal in scope. A recent consensus statement from a group of leading international researchers working in the field of gerontology and loneliness also concluded that the current evidence base is dominated by “low-quality trials, small samples, a lack of theoretical frameworks or understanding of loneliness, diverse or undefined target groups, mixed measures of loneliness and short follow-up periods to assess longer term impact” [50]. Limits in the robustness of this evidence-base on what works may make it more difficult for both health service budget holders and local government authorities, who are responsible for public health in England, to determine how to combat loneliness.

In addition to wanting more information on what works, these policy makers will also be interested in the cost-effectiveness of investment in effective measures, that is whether the additional costs associated with intervention generate sufficient improvements in outcomes to justify the investment of what are very modest local public health budgets in England. Formal economic evaluations with loneliness alleviation as the primary outcome, however, remain rare. A recent systematic review of economic evaluations concluded that there is a paucity of economic evidence and that further research is needed to identify cost effective strategies [13].

However, this does not mean that potentially cost effective interventions are not available. An earlier review noted that even where loneliness was seen as the primary outcome in the effectiveness part of an evaluation, the economic analysis usually did not use a loneliness specific outcome [51]. An example of this is a study examining the cost-effectiveness of an organised community group singing to promote the mental health of socially isolated and lonely older women in England. This intervention was shown to be effective with a moderate 60% chance of being considered cost effective in the English context, but changes in quality of life rather than in loneliness were measured [52]. This makes it difficult to determine the specific changes in economic costs that can be attributed directly to changes in loneliness rather than other impacts of interventions. Another trial including a cost-effectiveness analysis in the UK that looked at structured professionally facilitated regular group activities for a community dwelling of older people did find evidence of significantly reduced loneliness in the intervention group at 24 months, but the primary focus of the study was on mental wellbeing where no effect was found [53]. Moreover, the authors of that study recognised the limitations of not proactively seeking to identify and reach older people at risk of poor mental wellbeing.

While a trial evidence on what works is limited, there is evidence on the relationship between loneliness and health care utilisation that might suggest a potential to avert some costs through loneliness alleviation. This is mainly drawn from longitudinal observational studies and can be used to inform models. For instance, a longitudinal analysis of panel data from the US Health and Retirement Study found an association between increased contacts with community-based doctors and chronic enduring loneliness, but no impact on hospital visits was seen [54]. A recent systematic review also suggests weak evidence that better social relationships may be associated with reduced hospital admissions and length of stay, but insufficient evidence on the association with use of primary care, outpatient and community services [55]. An analysis of data from the English Longitudinal Survey of Ageing suggests that, even when controlling for characteristics such as dementia diagnosis, loneliness independently is associated with a significantly increased risk of admission to long-term residential care [23]. This evidence continues to grow, for instance, another recent study making use of cross-sectional data from the UK’s Biobank reports a significant association between loneliness and both acute and chronic pain [56].

Given the limited evidence base, we constructed a Markov-decision analysis model to look at potential levels of cost-effectiveness that might be achieved, assuming that an effective intervention for loneliness alleviation is available and drawing on longitudinal studies suggesting that unaddressed loneliness may be associated with poorer levels of health, as well as more health and social care service utilisation. Our model suggests that if effective interventions can reduce and help sustain reduced levels of severe loneliness the value of any health and social care costs averted is unlikely to offset the cost of intervention. Our probabilistic sensitivity analysis revealed that just 3.5% of iterations of cost and effects would be cost saving from a health and social care perspective.

Nonetheless, an investment of £ 768 per loneliness free year gained may be a value that policy makers are willing to pay. Our illustrative real-world loneliness alleviation programme had a cost per client of £ 752. From a societal perspective, the cost-effectiveness ratio will be more favourable. We did not fully explore this in our analysis, although we did observe a reduction in the ICER to £ 695 when including the potential informal care costs associated with an increased risk of stroke and dementia in the severely lonely, as well as the positive value of volunteering by intervention clients who themselves become intervention volunteers.

The ICER will fall further when additional informal care costs for other conditions, as well as wider impacts such as lost opportunities to engage in volunteering and paid work beyond the retirement age, are considered. If there is scope to increase the relative proportion of service users who meet the criteria for severe rather than moderate levels of loneliness, the case for intervention may be strengthened further. Initiatives to increase the efficiency of programmes and therefore, reduce costs of delivery, will also improve cost-effectiveness. We also have conservatively not included any potential health benefits associated with a reduction in the moderate levels of loneliness, any such benefits would also improve the ICER.

Although we believe that our modelling analysis serves to illustrate the potential value of loneliness alleviation measures, there are key limitations that need to be addressed in further empirical studies. We have, for illustrative purposes, taken as our base scenario the observed aggregate reduction in loneliness levels routinely collected as part of the delivery of a loneliness alleviation programme in one area of southern England. There is no counterfactual group with which to compare incremental changes in loneliness, nor can we account for factors such as the availability of alternative community support services. Ideally, we would have liked to have drawn on effect size from a randomized controlled trial. Alternatively, in the future, it might be possible to create a counterfactual individual level simulated population estimate drawing on data from current longitudinal studies that look at ageing, as well as collecting data on loneliness and levels of community participation. That said, our estimates of effect may be conservative, a similar uncontrolled intervention in Wales reported aggregate reductions in loneliness which are double those used in this model [57].

We have also assumed that if an individual is severely lonely at the end of any Markov cycle this implies that they have been severely lonely for the entire year, but their loneliness levels may have varied considerably over this time period. We have also arbitrarily assumed that a year spent being moderately lonely has half the value of a year without loneliness. Another gap is a lack of knowledge on recovery from loneliness without intervention. One novel way in the future of exploring this issue might be for participants and a counterfactual group to use a mobile app to briefly indicate their current levels of loneliness on a regular basis. This could help explore the temporal nature of loneliness and how it ebbs and flows over time. This should be feasible as populations who are more familiar with these technologies get older. However, our purpose here is to illustrate the potential impacts of different levels of effect on loneliness rather than make the case for any individual programme and we have varied estimates of effect in both the univariate and probabilistic sensitivity analysis to account for uncertainty in our assumptions.

Another limitation is that we do not know enough on the causality of the relationship between loneliness and any impacts on health status and utilisation. It may be that the onset of poor health in part exacerbates pre-existing levels of loneliness in our study population. There may also be some overlapping of potential costs for closely related conditions such as coronary heart disease and strokes.

In terms of sustained effects consistent with the structure of the Reconnections programme that we have taken as our illustrative example, the model assumes that longer term nominal costs beyond the first year that may be charged for participating in group activities are borne by the participants themselves or the activity deliverer (such as a charity or community group). We have considered the impact of variation in sustained participation in the sensitivity analysis, but even nominal costs of £ 1 or £ 2 per week to a client may be a deterrent to long term participation in any programme in the real world.

Finally, in addition to recommending further research on both the effectiveness and cost-effectiveness of loneliness alleviation interventions, we also believe it is important to undertake work to map across levels of loneliness with validated instruments such as the UCLA [20] and De Jong Gierveld [58] loneliness scales to quality of life measures which are commonly in health economics, such as EuroQOL EQ-5D [59] or SF-6D [60]. One recent promising development is an analysis using both the De Jong Gierveld scale and the 12-Item Short Form Health Survey used in generating SF-6D scores. This study reported a significant inverse association between increasing loneliness in older people in five European countries and their physical and mental quality of life [61]. Further work could be undertaken to assess how these associations impact on utility weights used to calculate quality adjusted life years. Alternatively, as a standard, economic evaluations of these interventions could simply assess the impacts on quality of life in addition to impacts on loneliness. This would allow the cost per quality adjusted life to be calculated and thus allow an investment in measures to tackle loneliness to be compared with other public health and health care interventions.

## 5. Conclusions

Measures that reduce the levels of severe loneliness in older people may potentially be cost-effective depending on the value that society places on loneliness reduction. However, they are unlikely to be cost-saving even when making generous assumptions on the long-term sustained effects and potential adverse health and social care events averted. These promising findings justify an investment in well-designed empirical studies to determine the cost-effectiveness, as well as the willingness to participate in and remain engaged with measures, such as signposting and other social connection mechanisms, as a means of reducing loneliness in different contexts and settings.

## Figures and Tables

**Table 1:** Model parameters.

Input Parameter	Deterministic Value	Distribution in PSA	Source
Costs			
Accident and emergency department attendance (per visit). Assumed as the National Tariff Emergency Medicine 2019/20 VA Category 2 investigation with Category 2 treatment VB07Z	£ 163	Gamma	[27]
Ambulance (per emergency) using National Tariff 2018/19 ASSO2 See, Treat and Convey Tariff	£ 257	Gamma	[28]
Coronary Heart Disease (Annual)	£ 1637	Gamma	[29]
Dementia: Health and social care (Annual)	£ 19,383	Gamma	[30]
Dementia: Informal care (Annual)	£ 15,858	Gamma	[30]
Depression (Annual)	£ 961	Gamma	[31]
GP (per contact)	£ 39.23	Gamma	[32]
Hospital admission (per admission)	£ 631	Gamma	[32]
Intervention Cost (per client)	£ 752	Gamma	[33]
Residential care (private residential care home) (per week)	£ 691	Gamma	[32]
Self-harm treatment (per hospital presentation)	£ 895	Gamma	[34]
Stroke: Health and social care (annual)	£ 8833	Gamma	[35]
Stroke: Informal care (annual)	£ 22,589	Gamma	[35]
Incidence among over 65s			
Coronary heart disease	0.011	Beta	[36]
Dementia	0.008	Beta	[36]
NHS presenting depression	0.017	Beta	[37]
Stroke	0.003	Beta	[36]
Hospital presenting self-harm	0.001	Beta	[38]
Residential care admission	0.019	Beta	[39]
Increased relative risk of event if severely lonely			
Coronary heart disease	1.29	Log Normal	[5]
Dementia	1.58	Log Normal	[21]
Depression	1.23	Log Normal	[4]
GP Contact	1.86	Log Normal	[24]
Residential care admission	2.13	Log Normal	[23]
Stroke	1.32	Log Normal	[5]
Self-harm	17.37	Log Normal	[22]
Death	1.30	Log Normal	[6]
**Other**			
Percent of participants initially severely lonely at baseline with UCLA-4 scores >9	0.44	Beta	[33]
Percent of participants initially moderately lonely at baseline with UCLA-4 scores <9 and >6	0.56	Beta	[33]
Probability of becoming not lonely after an intervention	0.191	Beta	[33]
Annual probability of death in the over 65 population (assumed to be the rate for both genders aged 70)	0.015	Beta	[40]
Expected number of GP contacts per annum in over 65s	7.70	Gamma	[41]
Probability of dropout	0.89	Beta	[33]
Volunteering value (per hour) (Assumed equivalent to the national minimum wage for over 21s)	£ 0.21	Fixed	[42]
Probability of the participant becoming a permanent volunteer	0.003	Fixed	[43]
Annual Transition probabilities			
With Intervention			
Severely lonely to moderately lonely	0.444	Beta	[33]
Severely lonely to not lonely	0.187	Beta	[33]
Moderately lonely to severely lonely	0.351	Beta	[33]
Moderately lonely to not lonely	0.188	Beta	[33]
Not lonely to severely lonely	0.351	Beta	[33]
Not lonely to moderately lonely	0.446	Beta	[33]
Without intervention			
Severely lonely to moderately lonely	0.549	Beta	[33]
Severely lonely to not lonely	0	Beta	[33]
Moderately lonely to severely lonely	0.433	Beta	[33]
Moderately lonely to not lonely	0	Beta	[33]
Not lonely to severely lonely	0.433	Beta	[33]
Not lonely to moderately lonely	0.552	Beta	[33]

**Table 2:** Summary of cost-effectiveness findings (health and social care perspective).

	Loneliness Alleviation Intervention	No Intervention
Intervention	£ 669	£ 0
GP Visits	£ 1399	£ 1467
Depression	£ 62	£ 63
Self-Harm	£ 55	£ 65
Coronary Heart Disease	£ 72	£ 74
Stroke	£ 88	£ 90
Dementia	£ 649	£ 672
Other residential care	£ 3308	£ 3534
Other hospital contacts	£ 799	£ 818
Total Cost (95% CI)	£ 7131 (£ 5888, 8727)	£ 6783 (£ 5429, 8488)
Loneliness Free Years (95% CI)	1.77 (1.43, 2.19)	1.31 (1.15, 1.48)
ICER	£ 768 (£ 1632, 358)	

**Table 3:** Summary of cost-effectiveness findings (broader perspective including informal care costs for stroke and dementia).

	Loneliness Intervention	No Intervention
Intervention	£ 669	£ 0
GP Visits	£ 1399	£ 1467
Depression	£ 62	£ 63
Self-Harm	£ 55	£ 65
Coronary Heart Disease	£ 72	£ 74
Stroke	£ 315	£ 321
Dementia	£ 1180	£ 1222
Other residential care	£ 3308	£ 3534
Other hospital contacts	£ 799	£ 818
Additional volunteering	£ -10	£ 0
Total Cost (95% CI)	£ 7878 (£ 6595, 9481)	£ 7564 (£ 6179, 9304)
Loneliness Free Years (95% CI)	1.77 (1.43, 2.19)	1.31 (1.15, 1.48)
ICER	£ 695	

## Supplementary Materials

The following are available online at https://www.mdpi.com/1660-4601/18/4/1426/s1. Figure S1: Probabilistic sensitivity analysis, loneliness alleviation versus no action (partial societal perspective); Figure S2: Cost-effectiveness acceptability curve (partial societal perspective).
