# Supplementary material for: Modelling the Economic Impact of Reducing Loneliness in Community Dwelling Older People in England
Source: Int J Environ Res Public Health. 2021 Feb 3;18(4):1426. doi: 10.3390/ijerph18041426 (PMC7913744; doi:10.3390/ijerph18041426)
Supplement: Supplementary file 1 [file ijerph-18-01426-s001.pdf]

International Journal of
Environmental Research and Public Health

MDPI

Supplementary File

Additional probabilistic sensitivity analysis from partial societal perspective including informal care related to dementia and strokes.

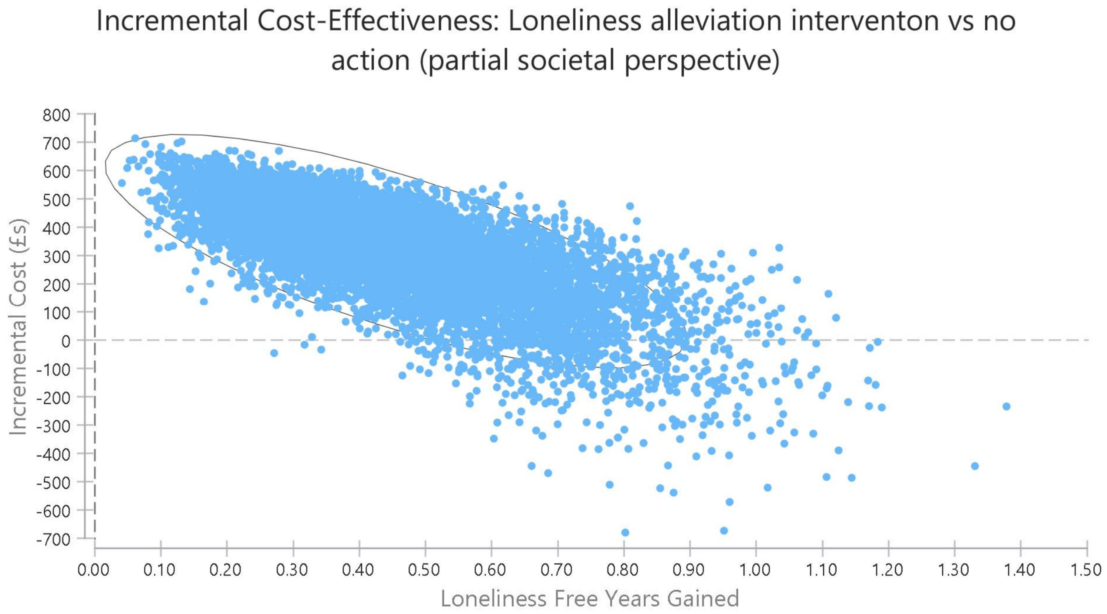

Figure S1. Probabilistic sensitivity analysis, loneliness alleviation versus no action (partial societal perspective).

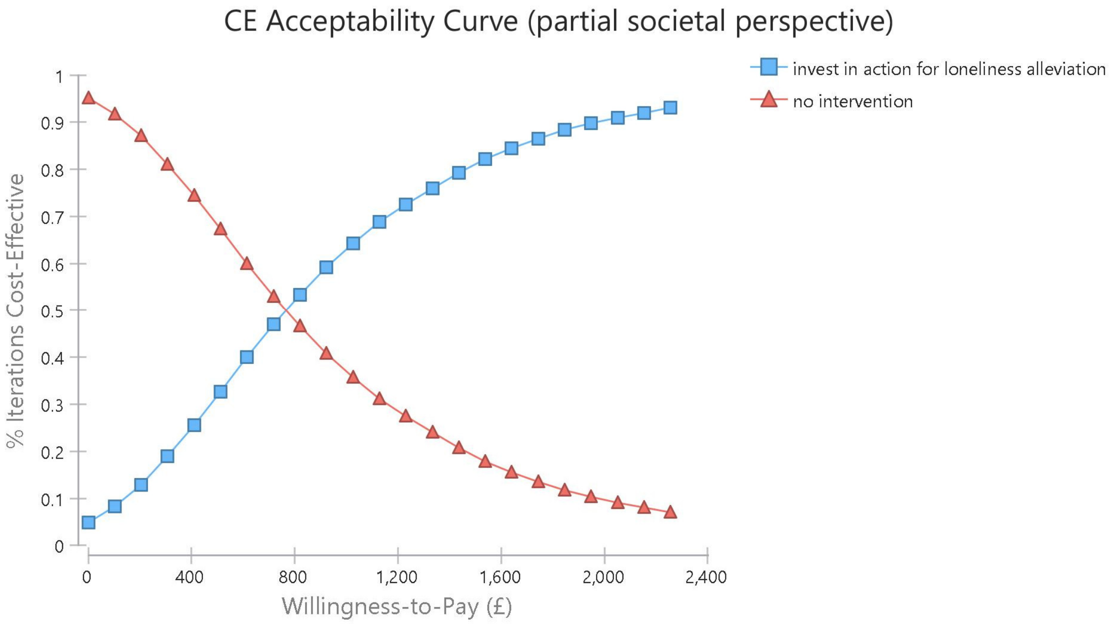

Figure S2. Cost-effectiveness acceptability curve (partial societal perspective)
